# Description of on-farm treatment compliance and risk factors for culling in sows

**DOI:** 10.1186/s40813-021-00238-7

**Published:** 2021-11-25

**Authors:** Magnus R. Campler, Jeremiah L. Cox, Heather L. Walker, Andréia G. Arruda

**Affiliations:** Department of Veterinary Preventive Medicine, College of Veterinary Medicine, The Ohio State University, 1920 Coffey Rd, Columbus, OH 43210 USA

**Keywords:** Antimicrobial resistance, Body condition score, Drug compliance, Sow treatments

## Abstract

**Background:**

In commercial pig farming, sick or injured sows are often treated by producers or hired staff. To date, limited quantitative data exists on treatment compliance and the possible effect on sow longevity post-treatment. The objective of the study was to quantify on-farm compliance of treatment selection, frequency, and dosage, as well as to investigate the association between body condition scores (BCS) and other sow-level factors on post-treatment cull risk.

**Results:**

On-farm treatment records, including culling reason or reason of death up to 6 months post-treatment, production records and sow characteristics were obtained for 134 sows over an 8-week period. Treatment compliance was based on the accuracy of recorded treatments compared to the herd veterinarian’s established treatment guidelines. Univariable and multivariable logistic regression models including treatment reason, treatment compliance, BCS, parity, production stage and production metrics, were constructed to investigate associations between those variables and sow culling or death. This study found low compliance for on-farm sow treatment protocols, with only 22.4% (30/134) of the sows receiving correct and complete treatment during the duration of the study. No effect of individual treatment components (drug, dosage, or frequency) on sow culling was observed. A trend for an interaction between treatment compliance and BCS was found, and parity and number of piglets born alive were identified as predictors for sow maintenance in the herd.

**Conclusions:**

On-farm sow treatment compliance was low, resulting in that approximately 80% of the enrolled sows were not treated according to existing guidelines. Non-compliance of treatment guidelines did not seem to affect the risk of culling in treated sows but may have prolonged any associated pain, recovery time and negatively impacted the sow welfare during that time period.

## Background

Sow culling due to disease and decision-based euthanasia are common in the swine industry [1]. Culling rates due to poor reproductive performance, injury or illness have been reported between 3.7 and 10% in US swine farms [1–3] with culling due to lameness being regarded as the most important welfare concern within the industry [4, 5].

In addition to sow welfare guidelines, removal decisions are a multifactorial equation based on farm economics; and often include factors such as sow parity, reproductive performance, poor leg conformity or lameness, and responsiveness to treatment [1, 4, 6]. Responsiveness to treatment is important as it gives sows a chance to recover before getting removed from the herd. In addition, rising global concerns regarding antimicrobial resistance (AMR) has created frameworks such as the Veterinary Feed Directive (VFD) to prevent any misuse or overuse of antibiotics critically important for human medicine [7–9]. The link between antibiotic use and AMR in multiple bacteria in swine has been well established prior to these directives in for instance, *Escherichia coli*, *Campylobacter* sp. and *Clostridium difficile* [10–12]. The increased concern for AMR in the swine industry has increased scrutiny of staff training, swine treatment protocols, administered dosages, and treatment timelines. Despite requirements of veterinary oversight, daily on-farm treatment decisions rarely rely on a swine veterinarian but rather on individual staff; and their individual-based experience and training may decide the sow’s health outcome. The lack of a residing veterinarian, proper training or protocols may create problems such as inconsistent treatment management and dosing of injectables [13, 14]. Therefore, to ensure proper treatment management whilst reducing the risk of AMR, it is imperative that sows at risk are accurately treated in a timely manner. The results derived from this study could be of practical use for farm managers, staff and other stakeholders to improve their treatment guidelines and oversight to ensure healthy sow herds.

The main hypotheses of the study were that on-farm compliance of drug selection, treatment frequency and dosage would be approximately 50%, and that sows with a low or high BCS as well as sows that did not receive treatments compliant with farm-specific guidelines would be at higher risk of culling or death within 6 months of treatment. A 6-month period was chosen to allow for any full or partial recovery as well as any effects on productivity to show that may prompt a culling decision. The objectives of this study were to (a) quantify on-farm compliance (based on farm-specific established guidelines) of drug selection, treatment frequency and dosage compliance in a sow farm over an 8-week period; and (b) investigate the association between sow BCS at treatment and risk factors (other than BCS) associated with the occurrence of culling or death within 6 months of treatment.

## Methods

### Study population and study design

This prospective observational cohort study was conducted between June to August of 2019 on a volunteering commercial 6,180-head farrow-wean swine farm in Ohio. The farm was split into two identical sides, each side containing eight farrowing rooms, and sharing one large gestation room. Each sow on the farm had a paper record (“sow card”), which was transcribed into electronic records in PigKnows (PigKnows LLC, Greeley, Colorado, USA). All sows were monitored daily for health problems and injuries by farm caretakers, per farm protocol. All caretakers on the farm had access to a treatment guide provided by the residing herd veterinarian. The treatment guide included common clinical diagnosis descriptions (e.g., lameness, mastitis, off-feed, and retained piglets) and appropriate injectable drugs and doses for common treatments. The treatment guide had information regarding two commonly used antibiotics; namely Excenel® (ceftiofur hydrochloride; Zoetis) and Lincomycin 300® (lincomycin; Durvet, Inc.), the analgesic Banamine-S® (flunixin meglumine; Merck Animal Health), and vitamin B12 (vitamin B12, Huvepharma, Inc.) as defined by the Food and Drug Administration (FDA). In addition, two extra label use drugs; namely Prevail™ (flunixin meglumine, MWI Veterinary Supply) and dexamethasone, that are currently not FDA approved for swine, were used on farm [9, 13, 15]. Additional treatments including hormones, anthelmintics or other antiparasitic drugs were not made available to researchers. Furthermore, detailed treatment regimens were kept confidential between the farm and the third-party veterinary company which provided these services.

For the purposes of the study, the farm was visited weekly for a period of eight weeks. During each visit, the research team examined all records on sow treatments that occurred during the past seven days. A sow was enrolled in the study if it had received treatment for a documented condition within the past seven days relative to the visit. For all enrolled sows, collected data variables were; production stage (lactation, bred, empty sows), parity, documented condition that triggered treatment (and severity, where applicable), treatment information (drug name, dose, and frequency), as well as the previous and most recent number of born alive and weaned piglets. Additionally, the BCS for each sow was assessed and recorded once by the visiting researchers on the day of the farm visit, using a qualitative scale: thin, ideal, and fat, based on a 1–5 BCS scale (adapted from [16]. Body scores 1 and 2 were considered ‘thin’, body score 3 was considered ‘ideal’, and body scores 4 and 5 were considered ‘fat’. Sow treated for lameness were assessed using the FeetFirst 4-point system; 0 = sow moves with little inducement, she is comfortable on all her feet; 1 = sow moves relatively easy, but visible signs of lameness are apparent in at least one leg, she is reluctant to bear weight on that leg but still moves easily from site to site in the barn; 2 = lameness is involved in one or more limbs, the sow exhibits compensatory behaviors such as dipping her head or arching her back; 3 = there is a real reluctance to walk and bear weight on one or more legs; it is difficult to move her from place to place on the farm (FeetFirst, Zinpro Corporation; Eden Prairie, MN, USA).

All recorded treatments received, including details on drug name, dose, and frequency, were cross-checked against the treatment guide, and four variables were created reflecting the use of the recommended treatments (treatment compliance criteria): drug compliance, drug dose compliance, drug frequency compliance, and the combination of compliance for drug, dose, and frequency (all correct). Those were each defined as binary variables, with a “1” representing an exact match between the treatment guide and the actual treatment, and a “0” representing one or more inconsistencies. A full list of the sow data collected is presented in Table 1. Electronic records for each sow were obtained at 6 months post-treatment to determine longevity in the herd (died, culled or remained in herd).

**Table 1:** Descriptive statistics for all sows (N = 134) separated by continuous and categorical variables

Continuous variables^1^	Category	Culled/died		Remained in herd		Total sows	
		Mean (SD)	Median (MAD)	Mean (SD)	Median (MAD)	Mean (SD)	Median (MAD)
Parity (n = 134)		4.3 (1.8)	5 (1.45)	3.2 (1.7)	3 (1.40)	3.8 (1.8)	4 (1.52)
Number of piglets born alive (n = 134)		12.1 (4.7)	13 (3.62)	13.4 (4.0)	14 (2.90)	12.7 (4.4)	14 (3.35)
Number of piglets weaned (n = 117)		12.9 (3.8)	12 (2.20)	12.4 (2.5)	12 (1.38)	12.7 (3.3)	12 (1.85)

Categorical/binary variables^1^		Culled/died N (%)	Remained in herd N (%)	Total sows N (%)
Body condition score (n = 111)	Thin	8 (13.6)	5 (9.6)	13 (11.7)
	Ideal	43 (72.9)	40 (76.9)	83 (74.8)
	Fat	8 (13.6)	7 (13.5)	15 (13.5)
Condition (n = 134)				
Lameness^*^ (n = 78)		35 (47.9)	43 (70.5)	78 (58.2)
	Severity 1	9 (25.7)	17 (39.5)	26 (33.3)
	Severity 2	15 (42.9)	15 (34.9)	30 (38.5)
	Severity 3	11 (31.4)	11 (25.6)	22 (28.2)
Mastitis (n = 18)		13 (17.8)	5 (8.2)	18 (13.4)
Reproductive disorder (n = 26)		19 (26.0)	7 (11.5)	26 (19.4)
Other (n = 12)^4^		6 (8.2)	6 (9.8)	12 (9.0)
Production Stage (n = 132)				
	Bred	27 (38.0)	34 (55.7)	61 (46.2)
	Lactation	37 (52.1)	20 (32.8)	57 (43.2)
	Empty	7 (9.9)	7 (11.5)	14 (10.6)
Drug compliance (n = 133)^2^		62 (84.9)	51 (83.6)	113 (84.3)
Dose compliance (n = 132)^2^		23 (31.5)	19 (32.2)	42 (31.8)
Frequency compliance (n = 133)^2^		56 (76.7)	45(75.0)	101 (75.9)
Overall compliance (n = 134)^3^		17 (23.3)	13 (21.3)	30 (22.4)

^1^The number of animals within each variable may differ based on completeness of obtained on-farm data

^2^Compliance was based on the residing veterinary recommendation, farm standard operation protocol and label compliance for each swine illness

^3^Overall compliance was defined as perfect compliance on drug, dose and frequency (all correct)

^4^This category includes infrequently reported health conditions (bleeding lesion, bacterial infection, off feed, diarrhea, non-specific inflammation, shoulder sores)

^*^No sow was scored a zero on the Zinpro lameness scale

### Statistical analysis

The minimum total sample size goal for this study was estimated at 98 (49 sows per group), based on the ability to detect a difference in proportions of 30% in culling for sows with ideal body score (30% culling risk) versus non-ideal body score (60% culling risk), by using a level of confidence of 0.95 and a power of 0.8. The calculations were performed using the Epitools Epidemiological Calculator (Sergeant, Ausvet) [17].

One hundred forty-four sows were initially enrolled in the study. However, due to unreliable records such as conflicting condition and treatment statements or missing data entry, ten sows had to be omitted from the final data set. The final number of sows available for statistical analyses was 134. Eleven different health conditions were recorded during the study but due to the low frequency, they were grouped into four representative categories: lameness (swollen foot, foot sores, foot abscess, N = 78), mastitis (N = 18), reproductive disorders (metritis, retained piglets, N = 26) or other (bleeding lesion, bacterial infection, off feed, diarrhea, non-specific inflammation, shoulder sores, N = 12), for analysis purposes. In addition, sows with a thin or fat BCS were merged into a ‘non-ideal’ category for comparison with sows with an ‘ideal’ BCS in further analyses. In all, 24 sows were missing BCS data due to undisclosed sow movements or inability to identify the sow (missing ear tag) at the time of the researcher’s arrival on farm.

**Fig. 1:**
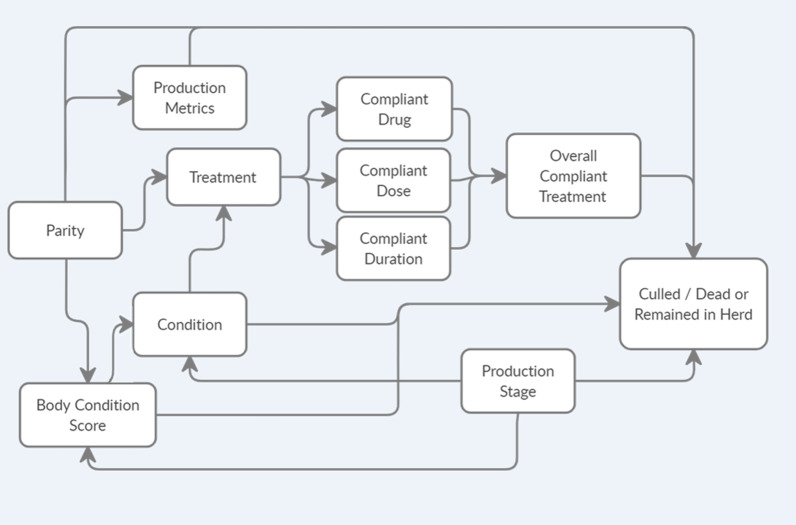
Causal diagram showing the outcome of interest (sows being culled or remaining in the herd, binary) and all predictor variables. Arrows point to possible associations. Predictors of interest: parity (continuous); condition (reason the animal was being treated, categorical); body condition score (categorical); production metrics (number of piglets born alive, number of piglets weaned, both continuous); compliance drug/ compliance dose/ compliance frequency/ overall compliance treatment (constructed by comparing reported use to veterinarian-recommended use, binary); and production stage (categorical). Treatment is listed to represent the decision to treat, which was an inclusion criteria for animals enrolled in the study

A multivariable logistic regression model was built using STATA/ IC 14.2 (College Station, TX; StataCorp LP). Model building steps included first checking for linearity between continuous variables of interest and the log of the outcome. For cases in which the linearity assumption was not met, the variable was categorized. Next, correlation was checked using the Spearman correlation coefficient, using a cut-off value of 0.80 to declare highly correlated variables. Univariable models were built, and variables with a *P*-value of 0.20 or lower were offered to a full multivariable model, along with any potential confounders identified through a causal diagram (Fig. 1). Variables considered for analysis included number of piglets born alive (representing the sow’s most recent reproductive cycle), previous number of piglets born alive (representing the sow’s last reproductive cycle), production stage (lactating, bred, or empty), health condition (lameness, mastitis, reproductive disorder, or other), body condition score (non-ideal or ideal), drug compliance (yes or no), drug dose compliance (yes or no), drug frequency compliance (yes or no), and the combination of compliance for drug, dose, and frequency (all correct; yes or no). All production data was obtained from farm records, any discrepancy in data from the original 134 enrolled sows, reflect missing data in the official farm records.

The final multivariable logistic model was built using a backwards stepwise approach. Confounders were determined as the removal of a variable changed the coefficient of other (s) variable (s) in the model by 20% or more and, in such cases, were retained in the model. Interactions were considered between our main variables of interest (BCS and treatment compliance) and all other variables retained in the models.

Statistical significance was declared at *P* < 0.05, and statistical trends were declared at 0.05 ≤ *P* < 0.10. Nested models were compared using Akaike’s Information Criterion (AIC) and the Bayesian Information Criterion (BIC), with lower numbers preferred for model selection. Lastly, model goodness-of-fit was evaluated using Hosmer–Lemeshow test.

## Results

### Descriptive statistics

**Fig. 2:**
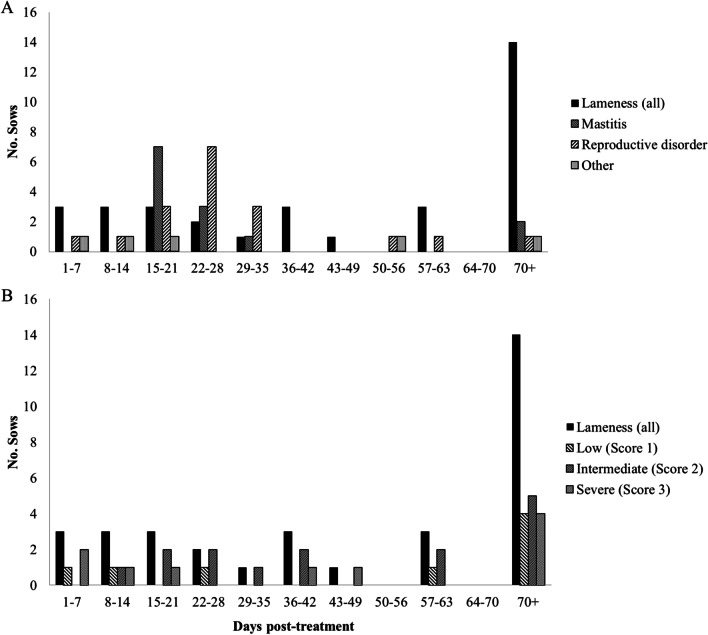
Distribution of study culled sow by condition and days after treatment. Distribution of culled sows treated for either lameness (N = 35), mastitis (N = 13), reproductive disorder (N = 19) or any other illness (N = 6) (**A**), and the distribution of clinically diagnosed lame cull sows (N = 69) and their respective lameness score (low: score 1; intermediate: score 2 or, severe: score 3, Feet First 4-point system, Zinpro) by removal time frame in days after the first treatment day (**B**)

Descriptive statistics for all variables captured in the study are presented in Table 1. A histogram showing the distribution of reasons for culling (referred to as “conditions”) for culled sows in relation to their removal time frame (days post-treatment) is depicted on Fig. 2A and the distribution of lameness scores for culled sows in relation to their removal time frame (days post-treatment) is depicted in Fig. 2B.

Overall, on-farm treatment compliance across the 134 treated sows for the combination of correct drug, treatment frequency, and dose regimen (all of these three criteria had to be correct as per the farm guidelines for a treatment to be considered “compliant”) was 22.4% (30/134).

The proportion of sows that received two out of three treatment compliance criteria correctly were 3.7% (5/134), 41.8% (56/134), 5.2% (7/134) for drug and dose, drug and frequency, and dose and frequency, respectively. Under half (45.5% (61/134)) of the treated sows only got one out of the three treatment components administered correctly and 3.7% (5/134) of all sows had all three treatment components administered incorrectly. Out of sows given an incorrect dose, 88.9% (80/90) were given a lower than recommended dose, and 11.1% (10/90) were given a higher than recommended dose.

### Statistical model results

Univariable modeling results showed that for a one-unit increase in parity, sows had 44% higher odds of getting culled (OR = 1.44, *P* = 0.001; Table 2). Sows diagnosed by farm personnel as having mastitis and reproductive disorders had higher odds of being culled compared to sows with lameness (OR = 3.19, *P* = 0.043 and OR = 3.33, *P* = 0.015, respectively). In addition, for a one-unit increase in the number of piglets born alive in the sows’ most recent reproductive cycle, the odds of culling tended to decrease by approximately 7% (OR = 0.93, *P* = 0.096; Table 2), however this tendency was not observed when investigating the effect of the previous litter (OR = 0.91, *P* = 0.115; Table 2). Lastly, sows that were lactating had higher odds of being culled compared to sows that were bred (OR = 2.33, *P* = 0.026, Table 2). No other significant results or tendencies were found (Table 2).

**Table 2:** Results from univariable logistic regression analysis investigating risk factors for sows to be culled or die after on-farm treatment

Variable		OR (SE)	95% CI	*P* value
Parity		1.43 (0.15)	1.16–1.77	0.001
Born alive (recent)		0.93 (0.04)	0.86–1.01	0.096
Born alive (past)		0.91 (0.06)	0.80–1.02	0.115
Number of weaned piglets		1.05 (0.06)	0.93–1.18	0.463
Production stage	Bred	Reference		
	Lactation	2.32 (0.88)	1.11–4.90	0.026
	Empty	1.26 (0.75)	0.39–4.03	0.698
Body condition score	Thin	1.49 (0.91)	0.45–4.93	0.515
	Ideal	Reference		
	Fat	1.06 (0.60)	0.35–3.20	0.913
Condition	Lameness	Reference		
	Mastitis	3.19 (1.83)	1.04–9.83	0.043
	Other	1.23 (0.76)	0.36–4.15	0.740
	Reproduction	3.33 (1.66)	1.26–8.84	0.015
Drug compliance^1^		0.99 (0.48)	0.38–2.59	0.991
Dose compliance^1^		0.97 (0.36)	0.46–2.02	0.932
Frequency compliance^1^		1.10 (0.45)	0.49–2.44	0.818
Overall compliance^2^		1.12 (0.47)	0.49–2.54	0.785

^1^Compliance was based on the residing veterinary recommendation, farm standard operation protocol and label compliance for each swine illness

^2^Overall compliance was defined as perfect compliance on drug, dose and frequency (all correct)

The final multivariable logistic regression model included parity, number of piglets born alive (for both most recent and last reproductive cycle), production stage, health condition, and the interaction between BCS and overall treatment compliance (Table 3). Due to the complicated relationship amongst the examined variables (Fig. 1), removal of any variable from the model changed other coefficients significantly and, therefore, all variables that were significant in univariable models were retained in the final model, even if they were not statistically significant. The Hosmer–Lemeshow test was not significant (*P* = 0.47), which indicated a good model fit.

**Table 3:** Results from final multivariable logistic regression analysis investigating risk factors for sows to be culled or die after on-farm treatment

Variable		OR (SE)	95% CI	*P* value
Parity		1.48 (0.26)	1.05–2.10	0.026
Born alive (current)		0.93 (0.06)	0.81–1.06	0.288
Born alive (past)		0.78 (0.07)	0.65–0.94	0.007
Production stage	Bred	Reference		
	Lactation	0.38 (0.34)	0.06–2.24	0.284
	Empty	1.21 (1.02)	0.23–6.32	0.822
Condition	Lameness	Reference		
	Mastitis	7.75 (10.36)	0.56–106.41	0.125
	Other	1.45 (1.39)	0.22–9.53	0.700
	Repro	4.12 (4.50)	0.49–35.00	0.195
Body condition score	Ideal	3.22 (2.24)	0.82–12.60	0.092
Overall treatment compliance^1^		4.53 (5.74)	0.38–54.38	0.234
Interaction between ideal BCS and overall treatment compliance		0.10 (0.12)	0.01–1.22	0.070

^1^Compliance was based on the residing veterinary recommendation, farm standard operation protocol and label compliance for each swine illness. Overall compliance was defined as perfect compliance on drug, dose and frequency (all correct)

Results from the final multivariable model showed that as parity increased by one unit, the odds of sows getting culled increased by approximately 50% (OR = 1.48, *P* = 0.026; Table 3) and as the number of piglets born alive in the sow’s last reproductive cycle increased by one piglet, the odds of sows to get culled decreased by approximately 20% (OR = 0.78, *P* = 0.007; Table 3).

**Fig. 3:**
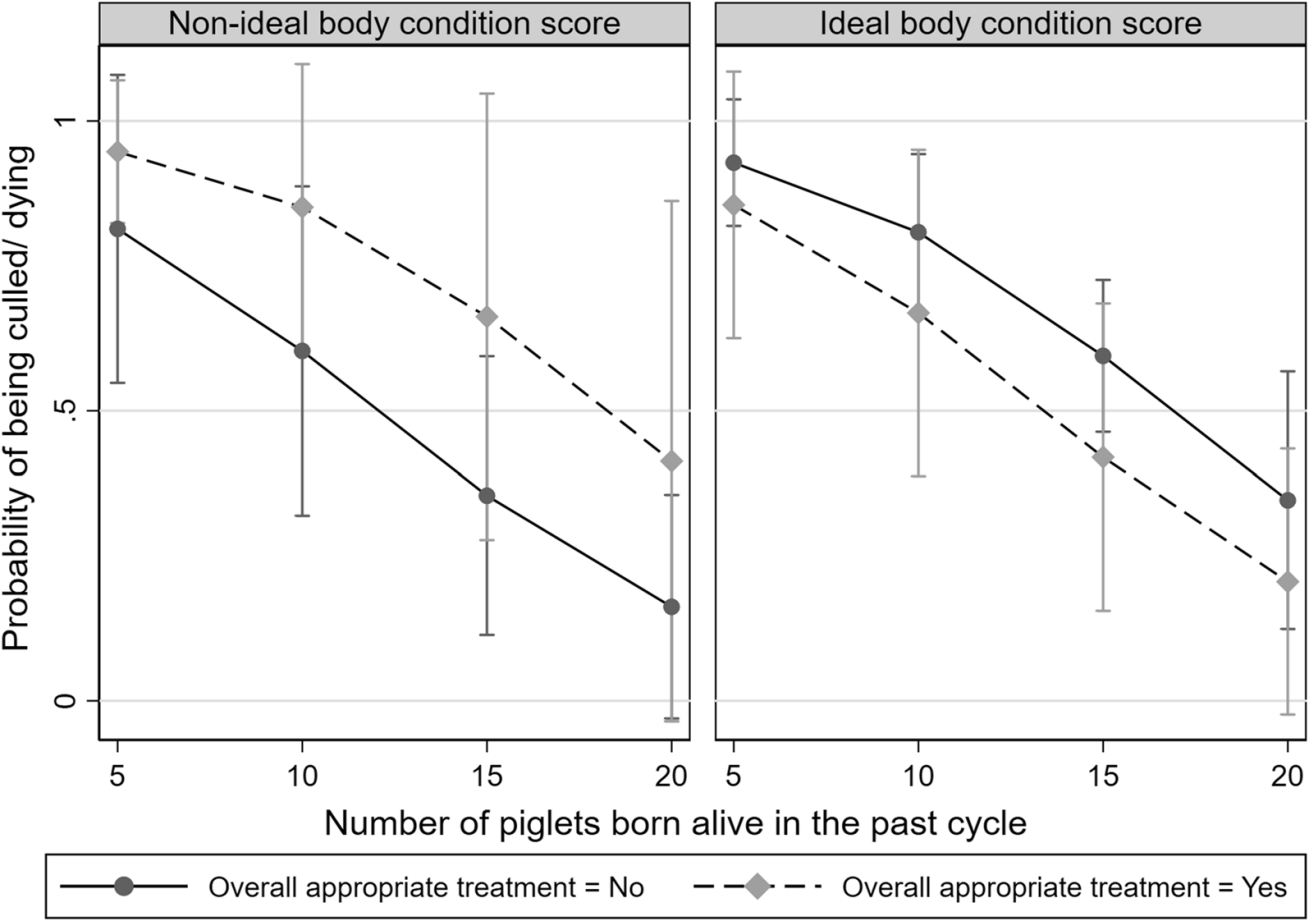
Interaction plot showing the relationship between treatment compliance and culling probability. Predictive margins with 95% CIs of sows (N = 111) overall compliant (correct drug, correct drug dose and correct frequency of treatment) or non-compliant treatment (either incorrect drug, dose, or frequency) with ideal or non-ideal body condition scores (BCS), (BCS = 2; N = 83) and (BCS = 1 or BCS = 3; N = 28) respectively, plotted over the numbers of piglets born alive in sow’s past reproductive cycle

The interaction between body condition and having received an appropriate treatment (all criteria met, including correct drug, dose and frequency) tended to be significant (*P* = 0.07). Figure 3 plots this interaction, using the number of piglets born alive in the sows’ past cycle as a third variable for illustration purposes. Figure 3 shows that, overall, the odds of sows being culled decreased as the number of piglets born alive in the past sow’s cycle increased. However, the nature of this association differed based on BCS and whether the sow received treatment according to the farm-specific treatment guidelines or not: for sows with a non-ideal BCS (high BCS or low BCS), the odds of being culled was greater for those receiving compliant treatments for all levels of number of piglets born alive, compared to sows not receiving compliant treatments. Conversely, for sows with ideal BCS, the probabilities of being culled were similar for sows receiving compliant treatments or not. In fact, sows not receiving per protocol treatment appeared to have a numerically higher probability of being culled across all numbers of piglets born alive described, as compared to sows not receiving appropriate treatment.

## Discussion

The main results showed that the on-farm treatment compliance was low, but that individual components of treatment compliance (correct drug, dosage or frequency, or their combinations) did not have any observed impact on sow culling or death. In contrast to our main hypotheses, this study found that only 22.4% of the treated sows received protocol-adherent treatment for their specific disease or illness. When protocol requirements were subdivided into compliance pairs (correct drug and treatment regimen (45.0%), correct drug and dose regimen (26.1%), or correct treatment frequency and dose regimen (27.6%)), only correct drug and treatment frequency was numerically near the hypothesized 50.0% compliance proportion. Despite the compliance outcome being lower than anticipated, there is no comparative data available for treatment compliance on sow farms. The low compliance may be indicative of the level of caretaker experience and training as these factors play an important role in how well they can perform their designated tasks [18]. Caretaker experience and training levels were not captured in this study, but the education level, ethnic and cultural background of the farm caretakers was diverse, which may have impacted the effectiveness of any on-farm training and in turn caused treatment inconsistencies or delays in timely culling [19]. The caretaker training aspect is important as non-compliance of treatment guidelines or management practices due to lack of training may lead to welfare risks through improper pain mitigation and prolonged disease conditions when under dosing, and increase antimicrobial resistance when overdosing [13, 20, 21].

Risk factors associated with increased odds of a sow being culled were parity, production stage, and the type of disease while the main factors associated with decreased odds of culling were the number of piglets born alive in the current and previous reproductive cycle. The proportion of sows culled from our study population was 54.5% which is consistent with earlier studies although culling rates above 70% have been reported [22, 23]. However, as our culling rate only applies to treated sows, a higher risk for culling was expected given the study population does not mirror all sows within a population. Although our study did not aim to confirm the link between the treatment and culling reason, confirmed culling rates due to lameness, reproductive disorders and mastitis fluctuate greatly in the literature [24–26].

In addition, we found that the sow’s odds of being culled increased by 50% as parity increased, and that approximately half of all culled sows (52.1%) were removed during the lactation stage of production. This could be a result of the increased risk of reproductive disorders and mastitis during the gestation and lactation period which were present in one-third of the sows in our study and comparable to earlier findings [27]. This may reflect an economic decision for the manager or owner, given that pregnant sows may still have a chance to farrow and wean a group of piglets before they are culled. Furthermore, the odds of being culled were reduced as the number of piglets born alive increased. Considering that producing viable weaned piglets is one of the most important characteristics for sows in a breeding herd, it is likely to influence culling decisions [4].

Surprisingly, our interaction showed that the majority of sows that were culled had an ideal body condition at time of treatment, while previous studies have shown increased health risks and, subsequently, increased cull rates, for non-ideal BCS [24, 28]. We hypothesize that our results might be explained by the fact that sows with a non-ideal BCS may be identified more easily and receive more timely and compliant treatment for illnesses unrelated to BCS, thus increasing the higher chances of recovering compared to sows with ideal body condition. In addition, as we defined non-ideal BCS as both thin and fat sows due low sample size, it is possible that the observed trend that non-ideal sows not receiving the overall appropriate treatment had a lower risk of being culled, may be driven by the fat pigs included in the group. As the model would not converge using the separate BCS categories, we recognize this issue, and the fact that this particular result may be counter-intuitive until we factor in the number of piglets born alive shown in Fig. 3. Thus, it is possible that the management decision of culling non-ideal sows were postponed due to a high viability of piglets for these particular sows.

Lameness was the most common ailment (47.9%) among sows that were ultimately removed, followed by reproductive disorders (26.0%) and mastitis (17.8%). Even though culling due to reproductive disorders has been reported to be more common than lameness and other common diseases [23], lameness is still one of the top concerns for animal welfare [4, 5]. Furthermore, it is possible that the farm in our study had an overarching issue with lameness and lameness treatment protocols, resulting in a higher lameness incidence compared to other swine farm. As no herd assessment baseline was established prior to the study, this factor remains unknown. The majority of sows (70.5%) remaining in the herd in our study were likewise treated for lameness, but the majority of those lameness cases were scored with a severity of 1, while sows that were culled were mostly scored as 2 or 3 (Table 1). The fact that culled sows were initially treated with higher severity scores could explain the higher potential for treatment failures, which could have aided in the removal decision.

Our study also had limitations. Firstly, the study population: this project was conducted on a single commercial farm during a short period of time, and only focused on sows rather than investigate the impact of treatment compliance on swine of different ages. Secondly, there is always a possibility of data discrepancies for both treatment and production data. For instance, perfect manual body condition and lameness scoring accuracy is hard to achieve, and even experienced scorers may score the same animal differently [29]. Thus, the obtained body condition scores for each sow were not validated or reassessed after the initial scoring and should therefore be interpreted with this in mind.

Additionally, it has been previously reported that official farm records do not always accurately reflect actual procedures or incidents that occur on-farm and that culling or death information provided in farm records not always match up to actual death records [28, 30, 31] To speculate, caretakers may have been unable to correctly diagnose certain illnesses or injuries. For instance, a misdiagnosed sow with osteochondrosis or a fracture may initially have been treated with antibiotics. Unless discovered later, this treatment may have been recorded as correct and in compliance for the perceived condition, but the sow would likely fail to recover and been prone to culling, a circumstance that would have been unpreventable and uncontrollable for the data collection. Thus, it is possible that farm records used for this study may contain errors that could have impacted the results of the study. In addition, no investigation of supportive therapy such as reallocation of sows to sick pens was included in this study.

There is always a possibility that farms that willingly and knowingly participate in a study attain a higher degree of detail and organization during the actual study period when researchers are on site. However, given that farm personnel were not aware of variables of interest or enrolled sows at the time of treatments; any deviation from normal procedures would have been minimal.

Future studies should include a larger number of farms, preferentially from different geographical regions, and include all ages of swine to give a better understanding of treatment compliance and record keeping across different stages of swine production in the industry, something that is currently lacking in the swine literature. Finally, future studies should also consider including an assessment of caretaker training, experience and attitudes as well as quantifying the recommended frequency for training to assure optimal animal care.

## Conclusions

Our study showed that on-farm sow treatment compliance, defined as caretakers following veterinarian-recommended protocols for specific drug, dose and frequency to treat specific health conditions, was low at approximately 22%. Despite low overall treatment compliance, sows were not at higher risk of culling post treatment regardless of received treatment compliance. Factors such as parity, number of piglets born and body condition score were associated to a higher degree in determining the risk of treated sows to be culled or remain in the herd post treatment. Proper training for farm owners and farm staff is likely to be important to ensure treatment compliance, accurate farm records and a healthy sow herd, as non-compliance of treatment guidelines may negatively impact sow welfare. Further prospective studies should include a larger number of herds and animals not only to establish the true prevalence of treatment non-compliance in the swine industry, but also to increase the power in detecting causal relationships between a wide range of risk factors related to disease treatment and culling decisions.

## Data Availability

Not applicable.

## Abbreviations

AIC: Akaike’s Information Criterion
AMR: Anti-Microbial Resistance
BCS: Body Condition Score
BIC: Bayesian Information Criterion
FDA: Food and Drug Administration
OR: Odds ratio
VFD: Veterinary Feed Directive
